# Ironing out Neurodegeneration: New Class of Lipids Promotes Ferroptosis in Dopaminergic Neurons

**DOI:** 10.1021/acscentsci.3c00438

**Published:** 2023-05-12

**Authors:** Anastasiia Kostenko, Allegra T. Aron

**Affiliations:** Department of Chemistry and Biochemistry, University of Denver, Denver, Colorado 80210, United States

Neurodegenerative diseases significantly affect the global population; in fact, more than seven million Americans have either Alzheimer’s disease or Parkinson’s disease.^1,2^ Understanding molecular mechanisms of neurodegeneration is essential for disease prevention and treatment, and recent evidence suggests the role of ferroptosis in promoting neurodegeneration. In this issue of *ACS Central Science*, Alan, Lee, and co-workers report a new class of lipid metabolite that regulates neurodegeneration through ferroptosis.^3^

**Figure 1:**
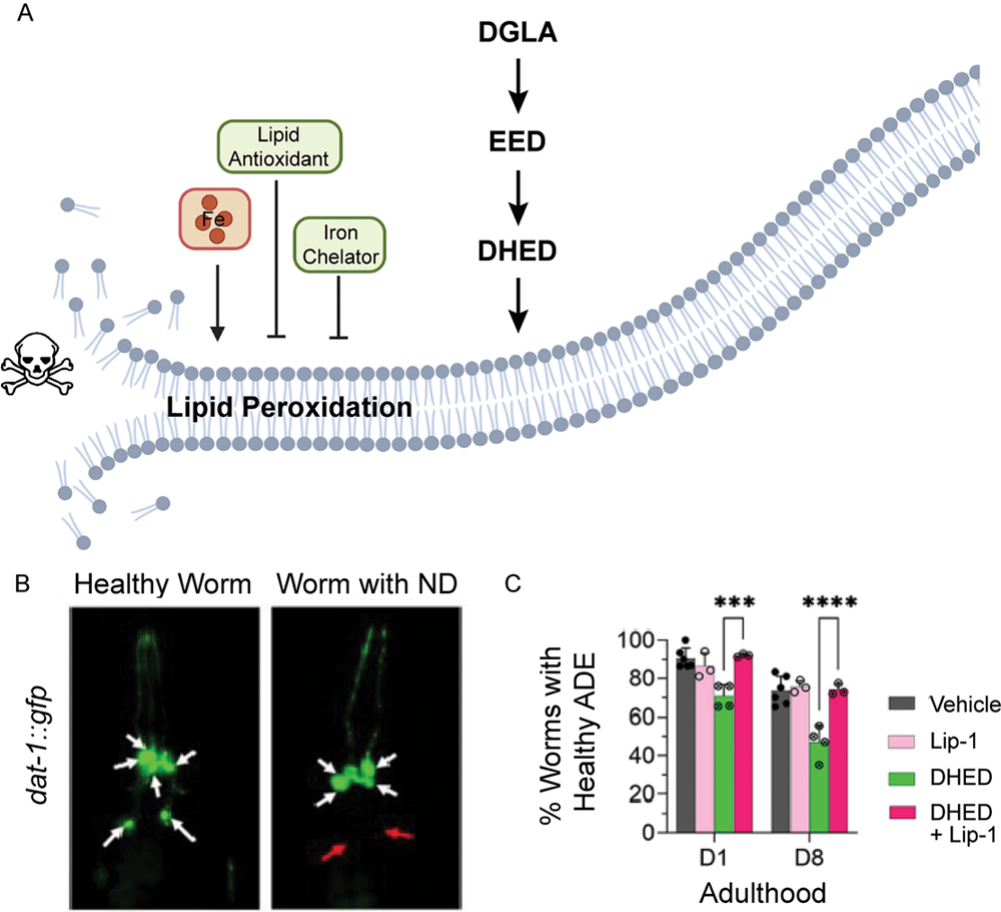
(A) The ferroptosis mechanism proposed in dopaminergic neurons in this study. Figure created with BioRender.com. (B) Dopaminergic neuron health can be monitored in worms with green fluorescent protein (GFP)-labeled dopaminergic neurons (Pdat-1::gfp worms), with healthy neurons highlighted by white arrows and degenerated neurons by red arrows. (C) The dihydroxyeicosadienoic acid (DHEH) metabolite produced downstream of ω-6 dihomo-gamma-linolenic acid (DGLA) results in cell death that can be rescued by two ferroptosis inhibitors—the lipid antioxidant Lip-1 (shown) and iron chelator 2,2′-bipyridine (not shown). B and C were reproduced with permission from ref (3). Copyright 2023 American Chemical Society.

Ferroptosis is a regulated mechanism of cell death that relies on iron and reactive oxygen species to catalyze lipid peroxidation preferentially in polyunsaturated fatty acids (PUFAs, Figure 1A).^4^ PUFAs are long-chain fatty acids that contain at least two double bonds and are classified as omega-3 (ω-3) or omega-6 (ω-6) based on the position of the first double bond relative to the methyl carbon. These lipids are obtained from diet and are particularly abundant in the brain with important roles in modulating membrane fluidity, receptor abundance and affinity, membrane-bound enzyme activity, along with signal transduction pathways.^5^ The beneficial effects of ω-3 PUFA supplementation on neurodegenerative disorders have been thoroughly studied, and an increased ω-3/ω-6 PUFA ratio has been linked to reduced risk of neurodegenerative disease, yet the roles of ω-6 PUFAs in neurodegeneration have not been established.^6^ Given that PUFAs are major substrates for lipid peroxidation in ferroptosis^7,8^ and that increased levels of lipid peroxidation and labile iron are hallmarks of neurodegeneration, some have hypothesized that ω-6 lipids could sensitize neurons to ferroptosis.^9,10^

If this hypothesis was correct, this study would contribute to the growing body of literature demonstrating ferroptosis as an important cell death mechanism in a number of diseases ranging from cancers to ischemia-reperfusion in the heart, brain, and kidneys.^11^ To test this hypothesis, the effect of four dietary ω-6 PUFAs and one ω-3 PUFA on fluorescently labeled neurons was monitored in *Caenorhabditis elegans* (Figure 1B). Interestingly, only ω-6 dihomo-gamma-linolenic acid (DGLA) was found to induce significant neurodegeneration. Dose-dependent and significant neurodegeneration was specifically observed in dopaminergic neurons, while mild to no neurodegeneration was observed in GABAergic, cholinergic, and glutaminergic neurons. DGLA was shown previously to induce ferroptosis in the *C. elegans* germline; to explore whether the observed degeneration in dopaminergic neurons was through ferroptosis or another cell death mechanism, control experiments were performed by cotreating neurons with DGLA alongside pharmacological inhibitors of ferroptosis (liproxstatin-1, Trolox, or 2,2′-bipyridine). Furthermore, DGLA was added to transgenic *C. elegans* strains containing nicotinamide adenine dinucleotide phosphate (NADPH)-oxidase (NOX) homologue loss of function mutant and ferritin knockout; NOX plays an important role in ferroptosis, while ferritin can protect cells from ferroptosis. In these experiments, pharmacological inhibitors of ferroptosis rescued neurodegeneration, whereas mutant neurons exhibited modulated neurodegeneration based on the genetic mutation. This study therefore showed that ω-6 DGLA promotes ferroptosis in dopaminergic neurons, and subsequent experiments indicated that this was mediated through a product of the DGLA metabolism.

DGLA is metabolized into epoxyeicosadienoic acid (EED) and dihydroxyeicosadienoic acid (DHED) by cytochrome P450 (CYP) and epoxide hydrolase (EH) enzymes, respectively. To determine whether DGLA itself or the EED and DHED metabolites promote neurodegeneration by ferroptosis, dopaminergic neurons were treated with chemically synthesized DHED or EED. These compounds caused more severe neurodegeneration than treatment with DGLA, and addition of ferroptosis inhibitors (Figure 1C) and EH inhibitors both alleviated the dopaminergic neurodegeneration. Taken together, these results suggest that DHED is the key mediator in the observed biology; this finding uncovers a novel mechanism of ferroptosis-mediated neurodegeneration that is modulated by endogenous levels of the DGLA metabolite. This study exhibits the power of chemical biology in elucidating complex mechanisms, as the authors used a multidisciplinary approach involving chemical synthesis, fluorescence imaging, genetic manipulation in a simple model organism, and biochemical enzyme inhibition to ultimately find the lipid that promotes ferroptosis.

The study provides novel mechanistic insights into the specific PUFAs involved in ferroptosis-mediated dopaminergic cell death. Interestingly, EH is upregulated in both Alzheimer’s and Parkinson’s disease, and recent reports show that inhibition of EH is neuroprotective; this study provides an example of how neurodegeneration could be controlled endogenously through modulation of DHED levels. By addressing key knowledge gaps, Alan, Lee, and co-workers provide insights into potential new therapeutic avenues for preventing ferroptosis-mediated neurodegeneration, i.e., modulation of dietary PUFAs, inhibitors of CYP-EH, or even potential use of ferroptosis inhibitors. We anticipate this finding will encourage further study into environmental factors mediating neurodegeneration, especially as ω-6 PUFAs are found in high levels in Western diets, and we hypothesize that chemical biology and metabolomics approaches can shed new light on lipid-promoted disease mechanisms. Understanding neurodegeneration through this lens of ferroptosis mediated by specific PUFAs adds another layer of complexity to the paradigm of neurodegeneration.
